# Stochastic synchronization of neuronal populations with intrinsic and extrinsic noise

**DOI:** 10.1186/2190-8567-1-2

**Published:** 2011-05-03

**Authors:** Paul C Bressloff, Yi Ming Lai

**Affiliations:** 1 Mathematical Institute, University of Oxford, 24-29 St. Giles', Oxford OX1 3LB, UK; 2 Department of Mathematics, University of Utah, 155 South 1400 East, Salt Lake City, Utah 84112, USA

## Abstract

We extend the theory of noise-induced phase synchronization to the case of a neural master equation describing the stochastic dynamics of an ensemble of uncoupled neuronal population oscillators with intrinsic and extrinsic noise. The master equation formulation of stochastic neurodynamics represents the state of each population by the number of currently active neurons, and the state transitions are chosen so that deterministic Wilson-Cowan rate equations are recovered in the mean-field limit. We apply phase reduction and averaging methods to a corresponding Langevin approximation of the master equation in order to determine how intrinsic noise disrupts synchronization of the population oscillators driven by a common extrinsic noise source. We illustrate our analysis by considering one of the simplest networks known to generate limit cycle oscillations at the population level, namely, a pair of mutually coupled excitatory (*E*) and inhibitory (*I*) subpopulations. We show how the combination of intrinsic independent noise and extrinsic common noise can lead to clustering of the population oscillators due to the multiplicative nature of both noise sources under the Langevin approximation. Finally, we show how a similar analysis can be carried out for another simple population model that exhibits limit cycle oscillations in the deterministic limit, namely, a recurrent excitatory network with synaptic depression; inclusion of synaptic depression into the neural master equation now generates a stochastic hybrid system.

## Introduction

Synchronous oscillations are prevalent in many areas of the brain including sensory cortices, thalamus and hippocampus [1]. Recordings of population activity based on the electroencephalogram (EEG) or the local field potential (LFP) often exhibit strong peaks in the power spectrum at certain characteristic frequencies. For example, in the visual system of mammals, cortical oscillations in the *γ *frequency band (20-70 Hz) are generated with a spatially distributed phase that is modulated by the nature of a visual stimulus. Stimulus-induced phase synchronization of different populations of neurons has been proposed as a potential solution to the binding problem, that is, how various components of a visual image are combined into a single coherently perceived object [2,3]. An alternative suggestion is that such oscillations provide a mechanism for attentionally gating the flow of neural information [4,5]. Neuronal oscillations may be generated by intrinsic properties of single cells or may arise through excitatory and inhibitory synaptic interactions within a local population of cells. Irrespective of the identity of the basic oscillating unit, synchronization can occur via mutual interactions between the oscillators or via entrainment to a common periodic stimulus in the absence of coupling.

From a dynamical systems perspective, self-sustained oscillations in biological, physical and chemical systems are often described in terms of limit cycle oscillators where the timing along each limit cycle is specified in terms of a single phase variable. The phase-reduction method can then be used to analyze synchronization of an ensemble of oscillators by approximating the high-dimensional limit cycle dynamics as a closed system of equations for the corresponding phase variables [6,7]. Although the phase-reduction method has traditionally been applied to deterministic limit cycle oscillators, there is growing interest in extending the method to take into account the effects of noise, in particular, the phenomenon of noise induced phase synchronization [8-15]. This concerns the counterintuitive idea that an ensemble of independent oscillators can be synchronized by a randomly fluctuating input applied globally to all of the oscillators. Evidence for such an effect has been found in experimental studies of oscillations in the olfactory bulb [11]. It is also suggested by the related phenomenon of spike-time reliability, in which the reproducibility of a single neuron's output spike train across trials is greatly enhanced by a fluctuating input when compared to a constant input [16,17].

In this paper we extend the theory of noise-induced phase synchronization to the case of a neural master equation describing the stochastic dynamics of an ensemble of uncoupled neuronal population oscillators with intrinsic and extrinsic noise. The master equation formulation of stochastic neurodynamics represents the state of each population by the number of currently active neurons, and the state transitions are chosen such that deterministic Wilson-Cowan rate equations [18,19] are recovered in an appropriate mean-field limit (where statistical correlations can be neglected) [20-23]. We will consider the particular version of the neural master equation introduced by Bressloff [23], in which the state transition rates scale with the size *N *of each population in such a way that the Wilson-Cowan equations are obtained in the thermodynamic limit *N *→ ∞. Thus, for large but finite *N*, the network operates in a regime characterized by Gaussian-like fluctuations about attracting solutions (metastable states) of the mean-field equations (at least away from critical points), combined with rare transitions between different metastable states [24]. (In contrast, the master equation of Buice *et. al*. assumes that the network operates in a Poisson-like regime at the population level [21,22]). The Gaussian-like statistics can be captured by a corresponding neural Langevin equation that is obtained by carrying out a Kramers-Moyal expansion of the master equation [25]. One motivation for the neural master equation is that it represents an intrinsic noise source at the network level arising from finite size effects. That is, a number of studies of fully or sparsely connected integrate-and-fire networks have shown that under certain conditions, even though individual neurons exhibit Poisson-like statistics, the neurons re asynchronously so that the total population activity evolves according to a mean-field rate equation [26-30]. However, formally speaking, the asynchronous state only exists in the thermodynamic limit *N *→ ∞, so that fluctuations about the asynchronous state arise for finite *N *[31-34]. (Finite-size effects in IF networks have also been studied using linear response theory [35]).

The structure of the paper is as follows. First, we introduce the basic master equation formulation of neuronal population dynamics. We reduce the master equation to a corresponding neural Langevin equation and show that both intrinsic and extrinsic noise sources lead to multiplicative white noise terms in the Langevin equation. We then consider an ensemble of uncoupled neuronal populations each of which evolves according to a neural master equation. We assume that each population supports a stable limit cycle in the deterministic or mean-field limit. We apply stochastic phase reduction and averaging methods to the corresponding system of neural Langevin equations, following along similar lines to Nakao *et al *[12], and use this to determine how independent intrinsic noise disrupts synchronization due to a common extrinsic noise source. (Previous studies have mostly been motivated by single neuronal oscillator models, in which both the independent and common noise sources are extrinsic to the oscillator. In contrast, we consider a stochastic population model in which the independent noise sources are due to finite size effects intrinsic to each oscillator). We then apply our analysis to one of the simplest networks known to generate limit cycle oscillations at the population level, namely, a pair of mutually coupled excitatory (*E*) and inhibitory (*I*) subpopulations [36]. A number of modeling studies of stimulus-induced oscillations and synchrony in primary visual cortex have taken the basic oscillatory unit to be an E-I network operating in a limit cycle regime [37,38]. The E-I network represents a cortical column, which can synchronize with other cortical columns either via long-range synaptic coupling or via a common external drive. In the case of an E-I network, we show how the combination of intrinsic independent noise and extrinsic common noise can lead to clustering of limit cycle oscillators due to the multiplicative nature of both noise sources under the Langevin approximation. (Clustering would not occur in the case of additive noise). Finally, we show how a similar analysis can be carried out for another important neuronal population model that exhibits limit cycle oscillations in the deterministic limit, namely, an excitatory recurrent network with synaptic depression; such a network forms the basis of various studies of spontaneous synchronous oscillations in cortex [39-43]. We also highlight how the inclusion of synaptic depression into the master equation formulation leads to a novel example of a stochastic hybrid system [44].

## Neural Langevin equation

Suppose that there exist *M *homogeneous neuronal subpopulations labeled *i *= 1, ..., *M*, each consisting of *N *neurons^a^. Assume that all neurons of a given subpopulation are equivalent in the sense that the pairwise synaptic interaction between a neuron of subpopulation *i *and a neuron of sub-population *j *only depends on *i *and *j*. Each neuron can be in either an active or quiescent state. Let *N_i_*(*t*) denote the number of active neurons at time *t*. The state or configuration of the full system (network of subpopulations) is then specified by the vector **N**(*t*) = (*N*_1_(*t*), *N*_2_(*t*), ..., *N_M_*(*t*)), where each *N_i_*(*t*) is treated as a discrete stochastic variable that evolves according to a one-step jump Markov process. Let *P*(**n**, *t*) = Prob[**N**(*t*) = **n**] denote the probability that the full system has configuration **n **= (*n*_1_, *n*_2_, ..., *n_M_*) at time *t, t *> 0, given some initial distribution *P*(**n**, 0). The probability distribution is taken to evolve according to a master equation of the form [20-23](1)

with boundary condition *P*(**n**, *t*) ≡ 0 if *n_i _*= -1 or *n_i _*= *N *+ 1 for some *i*. Here **e***_k _*denotes the unit vector whose *k*th component is equal to unity. The corresponding transition rates are chosen so that in the thermodynamic limit *N *→ ∞ one recovers the deterministic Wilson-Cowan equations [18,19] (see below):(2)

where *α_k _*are rate constants, *w_kl _*is the effective synaptic weight from the *l*th to the *k*th population, and *I_k _*are external inputs. The gain function *F *is taken to be the sigmoid function(3)

with gain *γ *and maximum firing rate *F*_0_. (Any threshold can be absorbed into the external inputs (*I_k_*). Equation (1) preserves the normalization condition  for all *t *≥ 0. The master equation given by equations (1) and (2) is a phenomenological representation of stochastic neurodynamics [20,23]. It is motivated by various studies of noisy spiking networks which show that under certain conditions, even though individual neurons exhibit Poisson-like statistics, the neurons fire asynchronously so that the population activity can be characterized by fluctuations around a mean rate evolving according to a deterministic mean-field equation [26-29]. On the other hand, if population activity is itself Poisson-like, then it is more appropriate to consider an *N*-independent version of the master equation, in which *NF *→ *F *and **w**/*N *→ **w **[21,22]. The advantage of our choice of scaling from an analytical viewpoint is that one can treat *N*^-1 ^as a small parameter and use perturbation methods such as the Kramers-Moyal expansion to derive a corresponding neural Langevin equation [45].

Multiplying both sides of the master equation (1) by *n_k _*followed by a summation over all configuration states leads to(4)

where the brackets 〈...〉 denote a time-dependent ensemble averaging over realization of the stochastic dynamics, that is, 〈*f*(**n**)〉 = ∑**_n _***P*(**n**, *t*)*f*(**n**) for any function of state *f*(**n**). We now impose the mean-field approximation 〈*T_k,r_*(**n**)〉 ≈ *T_k,r_*(〈**n**〉), which is based on the assumption that statistical correlations can be neglected. Introducing the mean activity variables , we can write the resulting mean-field equations in the form(5)

Substituting for *T_k,r _*using equation (2) yields the deterministic Wilson-Cowan equations [19](6)

Strictly speaking, the mean-field description is only valid in the thermodynamic limit *N *→ ∞, and provided that this limit is taken before the limit *t *→ ∞ [24]. In this paper we are interested in the effects of intrinsic noise fluctuations arising from the fact that each neural subpopulation is finite.

Let us introduce the rescaled variables *x_k _*= *n_k_*/*N *and corresponding transition rates(7)

Equation (1) can then be rewritten in the form(8)

Treating *x_k _*as a continuous variable and Taylor expanding terms on the right-hand side to second order in *N*^-1 ^leads to the Fokker-Planck equation(9)

with ε = *N *^-1/2^,(10)

and(11)

The solution to the Fokker-Planck equation (9) determines the probability density function for a corresponding stochastic process **X**(*t*) with **X**(*t*) = (*X*_1_(*t*), ..., *X_M_*(*t*)), which evolves according to a neural Langevin equation of the form(12)

Here *W_k_*(*t*) denotes an independent Wiener process such that(13)

Equation (12) is the neural analog of the well known chemical Langevin equation [46,47]. (A rigorous analysis of the convergence of solutions of a chemical master equation to solutions of the corresponding Langevin equation in the mean-field limit has been carried out by Kurtz [48]). It is important to note that the Langevin equation (12) takes the form of an Ito rather than Stratonovich stochastic differential equation (SDE). This distinction will be important in our subsequent analysis.

The above neural Langevin equation approximates the effects of intrinsic noise fluctuations when the number *N *of neurons in each sub-population is large but finite. It is also possible to extend the neural Langevin equation to incorporate the effects of a common extrinsic noise source. In particular, suppose that the external drive *I_k _*to the *k*th sub-population can be decomposed into a deterministic part and a stochastic part according to(14)

where *h_k _*is a constant input and *ξ*(*t*) is a white noise term, which is assumed to be common to all the neural subpopulations; the level of extrinsic noise is given by the dimensionless quantity *σ *and . Substituting for *I_k _*in equation (7) and assuming that *σ *is sufficiently small, we can Taylor expand *Ω*_*k*,1 _to first order in *σ *to give(15)

Carrying out a corresponding expansion of the drift function *A_k_*(**x**) then leads to the extended neural Langevin equation(16)

where(17)

and *dW*(*t*) = *ξ*(*t*)*dt *is an additional independent Wiener process that is common to all subpopulations. We now have a combination of intrinsic noise terms that are treated in the sense of Ito, and an extrinsic noise term that is treated in the sense of Stratonovich. The latter is based on the physical assumption that external sources of noise have finite correlation times, so that we are considering the external noise to be the zero correlation time limit of a colored noise process.

## Stochastic synchronization of an ensemble of population oscillators

In the deterministic limit (ε, *σ *→ 0) the neural Langevin equation (16) reduces to the mean-field equation (6). Suppose that the latter supports a stable limit cycle solution of the form  with x*(*t *+ *nT *) = x*(*t*) for all integers *t*, where *T *is the period of the oscillations.. The Langevin equation (16) then describes a noise-driven population oscillator. Now consider an ensemble of  identical population oscillators each of which consists of *M *interacting sub-populations evolving according to a Langevin equation of the form (16). We ignore any coupling between different population oscillators, but assume that all oscillators are driven by a common source of extrinsic noise. Introducing the ensemble label *μ*, *μ *= 1, ..., , we thus have the system of Langevin equations(18)

We associate an independent set of Wiener processes , *k *= 1...., *M *with each population oscillator (independent noise) but take the extrinsic noise to be given by a single Wiener process *W*(*t*) (common noise):(19)(20)(21)

Langevin equations of the form (18) have been the starting point for a number of recent studies of noise-induced synchronization of uncoupled limit cycle oscillators [9,11-15]. The one major difference from our own work is that these studies have mostly been motivated by single neuron oscillator models, in which both the independent and common noise sources are extrinsic to the oscillator. In contrast, we consider a stochastic population model in which the independent noise sources are due to finite size effects intrinsic to each oscillator. The reduction of the neural master equation (1) to a corresponding Langevin equation (16) then leads to multiplicative rather than additive noise terms; this is true for both intrinsic and extrinsic noise sources. We will show that this has non-trivial consequences for the noise-induced synchronization of an ensemble of population oscillators. In order to proceed, we carry out a stochastic phase reduction of the full Langevin equations (18), following the approach of Nakao *et. al. *[12] and Ly and Ermentrout [15]. We will only sketch the analysis here, since further details can be found in these references. We do highlight one subtle difference, however, associated with the fact that the intrinsic noise terms are Ito rather than Stratonovich.

### Stochastic phase reduction

Introduce the phase variable *θ *∈ (-*π*, *π*] such that the dynamics of an individual limit cycle oscillator (in the absence of noise) reduces to the simple phase equation , where *ω *= 2*π*/*T *is the natural frequency of the oscillator and . The phase reduction method [6,7] exploits the observation that the notion of phase can be extended into a neighborhood ℳ ⊂ ℝ^2 ^of each deterministic limit cycle, that is, there exists an isochronal mapping *Ψ *: ℳ → [-*π*, *π*) with *θ *= *Ψ*(**x**). This allows us to define a stochastic phase variable according to *Θ*^(*μ*)^(*t*) = *Ψ*(**X**^(*μ*)^)(*t*)) ∈ [-*π*, *π*) with **X**^(*μ*)^(*t*) evolving according to equation (18). Since the phase reduction method requires the application of standard rules of calculus, it is first necessary to convert the intrinsic noise term in equation (18) to a Stratonovich form [25,49]:(22)

The phase reduction method then leads to the following Stratonovich Langevin equations for the the stochastic phase variables *Θ*^(*μ*)^, *μ *= 1, ..., [9,12,14]:(23)

Here *Z_k _*(*θ*) is the *k*th component of the infinitesimal phase resetting curve (PRC) defined as(24)

with . All the terms multiplying *Z_k_*(*θ*) are evaluated on the limit cycle so that(25)

It can be shown that the PRC is the unique 2*π*-periodic solution of the adjoint linear equation [7](26)

where *A_lk _*= *∂A_l_*/*∂x_k_*, which is supplemented by the normalization condition . The PRC can thus be evaluated numerically by solving the adjoint equation backwards in time. (This exploits the fact that all non-zero Floquet exponents of solutions to the adjoint equation are positive). It is convenient to rewrite equation (23) in the more compact form(27)

where(28)

In order to simplify the analysis of noise-induced synchronization, we now convert equation (27) from a Stratonovich to an Ito system of Langevin equations:(29)

where {*ζ*^(*μ*)^(***Θ***, *t*)} are correlated Wiener processes with . That is,(30)

with 〈*dζ*^(*μ*)^(***Θ***, *t*)〉 = 0 and 〈*dζ*^(*μ*)^(***Θ***, *t*)*dζ*^(*ν*)^(***Θ***, *t*)〉 = *C*^(*μν*)^(***Θ***)*dt*, where *C*^(*μν*)^(***θ***) is the equal-time correlation matrix(31)

The drift term  is given by(32)

with(33)

It follows that the ensemble is described by a multivariate Fokker-Planck equation of the form(34)

Equation (34) was previously derived by Nakao *et al *[12] (see also [15]). Here, however, there is an additional contribution to the drift term  arising from the fact that the independent noise terms appearing in the full system of Langevin equations (18) are Ito rather than Stratonovich, reflecting the fact that they arise from finite size effects.

### Steady-state distribution for a pair of oscillators

Having obtained the FP equation (34), we can now carry out the averaging procedure of Nakao *et. al. *[12]. The basic idea is to introduce the slow phase variables  according to *θ^μ ^*= *ωt *+ *ψ^μ ^*and set *Q*(*ψ*,*t*) = *P*({*ωt *+ *θ*^(*μ*)^}, *t*). For sufficiently small ε and *σ*, *Q *is a slowly varying function of time so that we can average the Fokker-Planck equation for *Q *over one cycle of length *T *= 2*π */*ω*. The averaged FP equation for *Q *is thus [12](35)

where(36)

and(37)

with(38)

Following Nakao *et. al. *[12] and Ly and Ermentrout [15], we can now investigate the role of finite size effects on the noise-induced synchronization of population oscillators by focussing on the phase difference between two oscillators. Setting  = 2 in equation (35) gives

Performing the change of variables

and writing *Q*(*ψ*^(1)^, *ψ*^(2)^, *t*) = *Ψ*(*ψ*, *t*)*Φ*(*ϕ*, *t*) we obtain the pair of PDEs

and

These have the steady-state solution(39)

where *Γ*_0 _is a normalization constant.

A number of general results regarding finite size effects immediately follow from the form of the steady-state distribution *Φ*_0_(*ϕ*) for the phase difference *ϕ *of two population oscillators. First, in the absence of a common extrinsic noise source (*σ *= 0) and ε > 0,*Φ*_0_(*ϕ*) is a uniform distribution, which means that the oscillators are completely desynchronized. On the other hand, in the thermodynamic limit *N *→ ∞ we have ε = *N *^-1/2 ^→ 0 so that the independent noise source vanishes. The distribution *Φ*_0_(*ϕ*) then diverges at *θ *= 0 while keeping positive since it can be shown that *g*(0) ≥ *g*(*θ*) [12]. Hence, the phase difference between any pair of oscillators accumulates at zero, resulting in complete noise-induced synchronization. For finite *N*, intrinsic noise broadens the distribution of phase differences. Taylor expanding *g*(*ϕ*) to second order in *ϕ *shows that, in a neighbourhood of the maximum at *ϕ *= 0, we can approximate *Φ*_0_(*ϕ*) by the Cauchy distribution

for an appropriate normalization . Thus the degree of broadening depends on the ratio

The second general result is that the functions *α*(*θ*) and *β_k_*(*θ*) that determine g(*ϕ*) and h(*ϕ*) according to equations (38) are nontrivial products of the phase resetting curves *Z_k_*(*θ*) and terms *a_k_*(*θ*), *b_k_*(*θ*) that depend on the transition rates of the original master equation, see equations (17), (25) and (28). This reflects the fact that both intrinsic and extrinsic noise sources in the full neural Langevin equation (18) are multiplicative rather than additive. As previously highlighted by Nakao *et al *[12] for a Fitzhugh-Nagumo model of a single neuron oscillator, multiplicative noise can lead to additional peaks in the function *g*(*ϕ*), which can induce clustering behavior within an ensemble of noise-driven oscillators. In order to determine whether or not a similar phenomenon occurs in neural population models, it is necessary to consider specific examples. We will consider two canonical models of population oscillators, one based on interacting sub-populations of excitatory and inhibitory neurons and the other based on an excitatory network with synaptic depression.

## Excitatory-inhibitory (E-I) network

### Deterministic network

**Figure 1:**
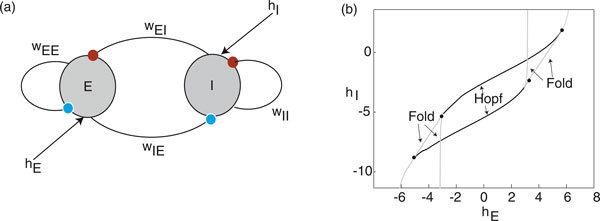
**Deterministic E-I network**. (a) Schematic diagram of network architecture. (b) Phase diagram of two-population Wilson-Cowan model (40) for fixed set of weights *w_EE _*= 11.5, *w_IE _*= -*w_EI _*= 10, *w_II _*= -2. Also *F*_0 _= *γ *= 1. The dots correspond to Takens-Bogdanov bifurcation points.

Consider a network model consisting of an excitatory subpopulation interacting with an inhibitory subpopulation as shown in Figure 1(a). The associated mean-field equation (6) for this so-called E-I network reduces to the pair of equations (dropping the bar on  and setting *M *= 2)(40)

Where *α_E,I _*= *α *= 1 for simplicity. (We interpret *α*^-1 ^as a membrane time constant and take *α*^-1 ^= 10 msec in physical units). Also note that *w_EE_*, *w_IE _*≥ 0 and *w_EI_*, *w_II _*≤ 0. The bifurcation structure of the Wilson-Cowan model given by equations (40) has been analyzed in detail else-where [36]. An equilibrium () is obtained as a solution of the pair of equations(41)

Suppose that the gain function *F *is the simple sigmoid *F *(*u*) = (1 + *e*^-*u*^)^-1^, that is, *F*_0 _= 1 and *γ *= 1 in equation (3). Using the fact that the sigmoid function then satisfies *F*' = *F*(1 - *F*), the Jacobian obtained by linearizing about the fixed point takes the simple form

An equilibrium will be stable provided that the eigenvalues *λ*_± _of **J **have negative real parts, where

This leads to the stability conditions **Tr J **< 0 and Det **J **> 0. For a fixed weight matrix **w**, we can then construct bifurcation curves in the ()-plane by imposing a constraint on the eigenvlaues *λ*_±_. For example, the constraint

with Det **J **> 0 determines Hopf bifurcation curves where a pair of complex conjugate eigenvalues crosses the imaginary axis. Since the trace is a quadratic function of , we obtain two Hopf branches. Similarly, the constraint Det **J **= 0 with Tr **J **< 0 determines saddle-node or fold bifurcation curves where a single real eigenvalue crosses zero. The saddle-node curves have to be determined numerically, since the determinant is a quartic function of . Finally, these bifurcation curves can be replotted in the (*h_E_*, *h_I_*)-plane by numerically solving the fix point equations (41) for fixed **w**. An example phase diagram is shown in Figure 1(b).

**Figure 2:**
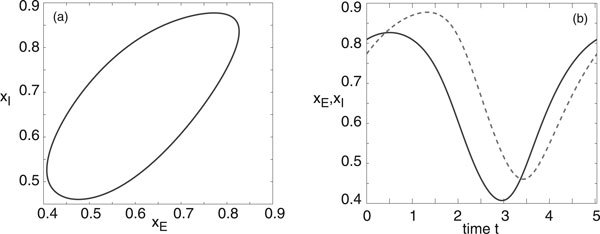
**Limit cycle in a deterministic E-I network with parameters *w_EE _*= 11.5, *w_IE _*= -*w_EI _*= 10, *w_II _*= -2, *h_E _*= 0 and *h_I _*= -4**. Also *F*(*u*) = 1/(1 + *e*^-*u*^). (a) Limit cycle in the (*x_E_*, *x_I_*)-plane. (b) Trajectories along the limit cycle for *x_E_*(*t*) (solid curve) and *x_I_*(*t*) (dashed curve).

**Figure 3:**
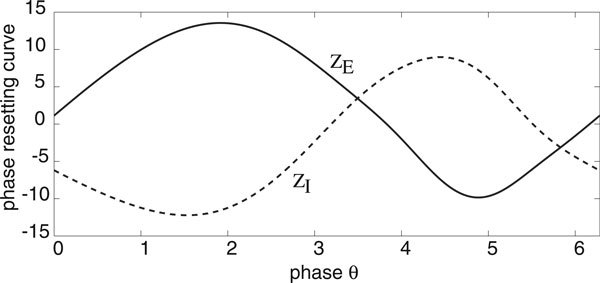
**Components *Z_E _*and *Z_I _*of phase resetting curve for an E-I network supporting limit cycle oscillations in the deterministic limit**. Same network parameter values as Fig. 2.

We will assume that the deterministic E-I network operates in a parameter regime where the mean-field equations support a stable limit cycle. For concreteness, we take a point between the two Hopf curves in Figure 1(b), namely, (*h_E_*, *h_I_*) = (0, -4). A plot of the limit cycle is shown in Figure 2 and the components *Z_E_*, *Z_I _*of the corresponding phase resetting curve are shown in Figure 3. Note that both components are approximately sinusoidal so that the E-I network acts as a type II oscillator.

### Stochastic network and noise-induced synchronization

**Figure 4:**
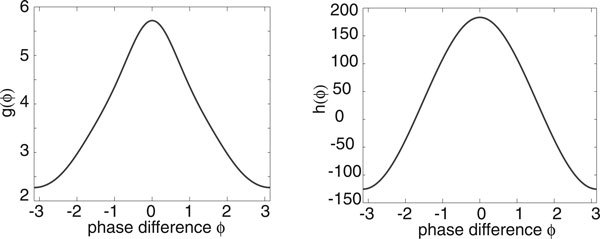
**Plot of periodic functions *g *and *h *for an E-I limit cycle oscillator with symmetric stochastic drive to excitatory and inhibitory populations (*χ_E _*= *χ_I _*= 1/2)**. Same network parameters as Fig. 2.

Let us now consider an ensemble of uncoupled stochastic E-I networks evolving according to the system of Langevin equations (18) for *M *= 2 and *k *= *E, I*. (More precisely, each E-I network evolves according to a master equation of the form (1). However, we assume that *N *is sufficiently large so that the master equation can be approximated by the corresponding Langevin equation. This was also checked explicitly in computer simulations). Having numerically computed the phase resetting curve (*Z_E_*, *Z_I_*) and the solution on the limit cycle for the deterministic E-I network, we can then compute the functions *g*(*ϕ*) and *h*(*ϕ*) of the stationary phase distribution *Φ*_0_(*ϕ*) according to equations (17), (25), (28) and (38). We plot these functions in Figure 4 for the parameter values of the limit cycle shown in Figure 2, assuming symmetric common noise to excitatory and inhibitory populations. That is, *χ_E _*= *χ_I _*= 1/2 in equation (17). It can be seen that the periodic function *g *is unimodal with *g*(0) ≥ *g*(*ϕ*) so that *Φ*_0_(*ϕ*) is also unimodal with a peak at *ϕ *= 0.

**Figure 5:**
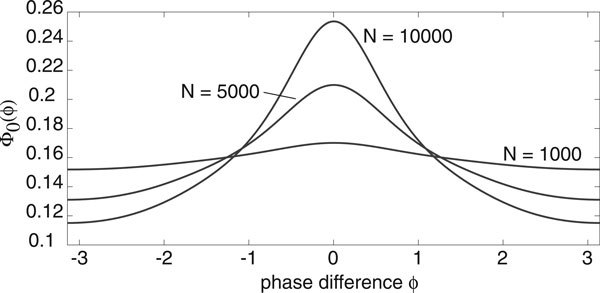
**Probability distribution of the phase difference between a pair of E-I oscillators as a function of system size *N *for fixed extrinsic noise *σ *= 0.08 with *g*, *h *given by Fig. 4**. Increasing *N *causes the curve to have a much sharper peak and much more synchronization.

**Figure 6:**
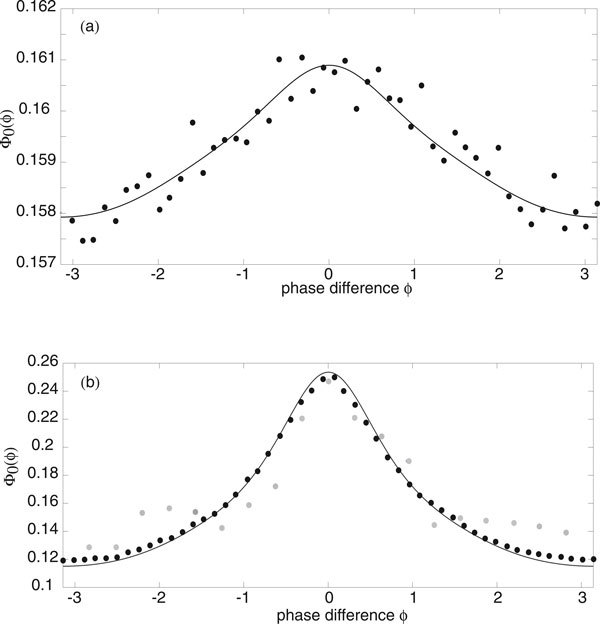
**Probability distribution of the phase difference between a pair of E-I oscillators as a function of extrinsic noise strength *σ *with *g*, *h *given by Fig. 4**. Solid curves are based on analytical calculations, whereas black (gray) filled circles correspond to stochastic simulations of the phase-reduced (planar) Langevin equations. (a) *N *= 10^5^, *σ *= 0.01. The curve is very flat, showing little synchronization. (b) *N *= 10^5^, *σ *= 0.08. Increasing *σ *causes the curve to have a much sharper peak and much more synchronization.

The width and height of the peak depend directly on the relative strengths of the intrinsic noise ε and extrinsic noise *σ*. This is illustrated in Figure 5 where the amplitude *σ *of the common signal is kept fixed but the system size *N *is varied. Increasing *N *effectively increases the correlation of the inputs by reducing the uncorrelated intrinsic noise, which results in sharper peaks and stronger synchronization, see also Marella and Ermentrout [13]. We find that there is good agreement between our analytical calculations and numerical simulations of the phase-reduced Langevin equations, as illustrated in Figure 6. We simulated the phase oscillators by using an Euler-Maruyama scheme on the Ito Langevin equation (29). A large number ℳ ≈ *O*(10^2^) of oscillators were simulated up to a large time *T *(obtained by trial and error), by which time their pairwise phase differences had reached a steady state. As we were comparing pairwise phase differences each simulation gave us  data points and we averaged over many simulations to obtain 10^6 ^data points for each diagram in Figure 6. These were then placed into 50 bins along [-*π*, *π*) and normalised. Also shown in Figure 6(b) are data points obtained from simulations of the full planar Langevin equations. Here computations were much slower so we only averaged over relatively few trials and thus the data is more noisy. Nevertheless a reasonable fit with the analytical distribution can still be seen.

**Figure 7:**
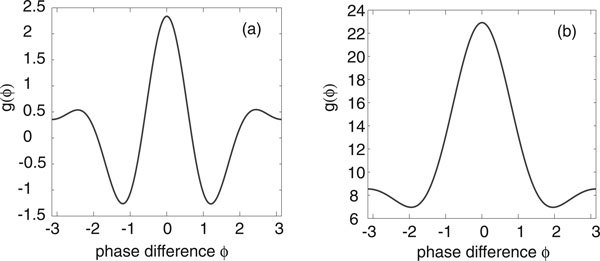
**Periodic function *g *with multiple peaks when there is an asymmetry in the common extrinsic noise source to the excitatory and inhibitory populations**. Other network parameters are as in Fig. 2. (a) *χ_E _*= 1/8, *χ_I _*= 7/8 so that common stochastic drive is mainly to the inhibitory population. (b) *χ_E _*= 7/8, *χ_I _*= 1/8 so that common stochastic drive is mainly to the excitatory population.

**Figure 8:**
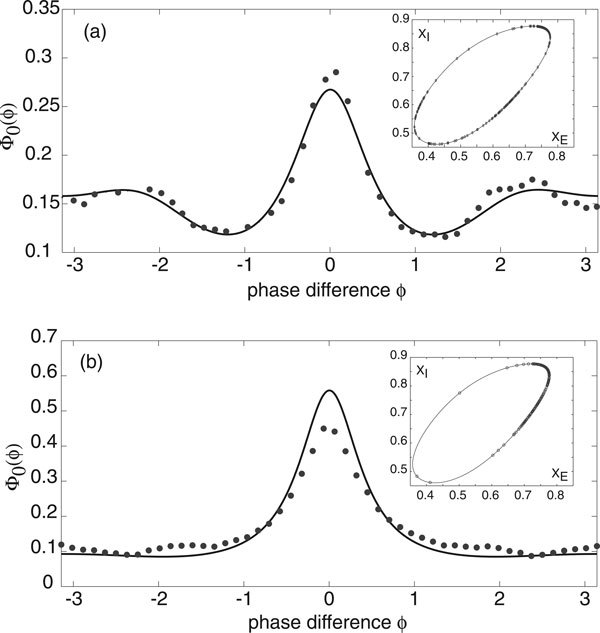
**Probability distribution of the phase difference between a pair of E-I oscillators when there is an asymmetry in the common extrinsic noise source to the excitatory and inhibitory populations**. Here *N *= 10^5^, *σ *= 0.01 and other network parameters are as in Fig. 2. Solid curves are based on analytical calculations, whereas black filled circles correspond to stochastic simulations of the phase-reduced Langevin equations. (a) *χ_E _*= 1/8, *χ_I _*= 7/8 so that common stochastic drive is mainly to the inhibitory population. (b) *χ_E _*= 7/8, *χ_I _*= 1/8 so that common stochastic drive is mainly to the excitatory population. The insets show instantaneous distributions of the oscillators on the limit cycle.

Nakao *et. al. *have previously shown that in the case of Stuart-Landau or Fitzhugh-Nagumo limit cycle oscillators with both uncorrelated and correlated extrinsic noise sources, parameter regimes can be found where the periodic function *g *has multiple peaks [12]. This can occur when higher harmonics of the phase resetting curve become dominant or when the common noise source is multiplicative. The presence of multiple peaks in *g *results in an ensemble of oscillators forming clustered states. Moreover, there are intermittent transitions between the clustered states induced by the uncorrelated noise. In the case of stochastic E-I limit cycle oscillators, we were unable to find a parameter regime where *g *develops multiple peaks when the common extrinsic noise source is the same for both excitatory and inhibitory populations, that is, *χ*_*E *_= *χ_I _*= 1/2 in equations (14) and (17). However, multiple peaks can occur when there is an asymmetry between the excitatory and inhibitory stochastic drives, as illustrated in Figure 7. The corresponding stationary distribution *Φ*_0_(*ϕ*) for the phase differences *ϕ *also develops additional peaks, see Figure 8. When the common stochastic input is mainly presented to the inhibitory population, we find a peak at *ϕ *= 0 and smaller peaks at *ϕ *= ±2*π*/3. Consequently, the ensemble of oscillators tend to cluster in three regions around the limit cycle as shown in the inset of Figure 8(a). On the other hand, when the stochastic drive is predominantly to the excitatory population, we find a much sharper peak at *ϕ *= 0 (compared to the symmetric case) and a small peak at *ϕ *= *π*. However, the latter does not contribute significantly to the dynamics, so that the oscillators are strongly synchronized.

## Excitatory network with synaptic depression

So far we have applied the stochastic phase reduction method to a two-population model consisting of mutually interacting excitatory and inhibitory populations. This E-I network is one of the simplest population models known to exhibit limit cycle oscillations in the deterministic limit, and forms the basic module in various studies of stimulus-induced oscillations and synchronization in visual cortex [37,38]. An even simpler population model known to exhibit limit cycle oscillations is a recurrent excitatory network with synaptic depression. For example, Tabak *et. al. *[39,40] have analyzed Wilson-Cowan mean-field equations representing a recurrent excitatory network with both slow and fast forms of synaptic depression, and used this to model the dynamics of synchronized population bursts in developing chick spinal cord. These burst oscillations are more robust in the presence of an extrinsic noise source or some form of spatial heterogeneity within the network [50,51]. An excitatory network with synaptic depression and extrinsic noise has also been used to model transitions between cortical Up and Down states [41-43]. Here we will show how our analysis of noise-induced synchronization of population oscillators based on a Langevin approximation of a neural master equation can be extended to take into account the effects of synaptic depression. In addition to the relevance of synaptic depression in the generation of neural oscillations, it is interesting from a mathematical perspective since the resulting master equation provides a novel example of a so-called stochastic hybrid system [44,52].

### Deterministic network

The mean-field equations for a homogeneous network with synaptic depression are taken to be of the form [53,54,39,43](42)

where we have set the membrane rate constant *α *= 1. The depression variable *q*(*t*) can be interpreted as a measure of available presynaptic resources at the population level, which are depleted at a rate *k*_-_*x*(*t*), which is proportional to the mean population activity *x*(*t*), and are recovered at a rate *k*_+_. A fixed point (x*, q*) of the mean-field equation satisfies q* = *k*_+_/(*k*_+ _+ *k*_-_x*) with x* given by

**Figure 9:**
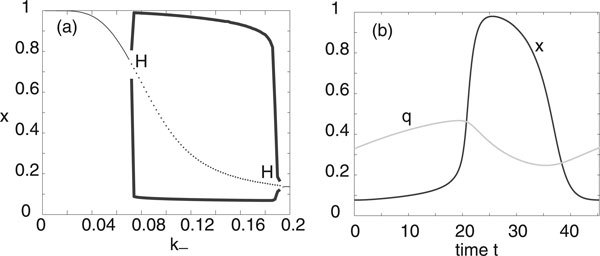
**Deterministic excitatory network with synaptic depression**. (a) Bifurcation diagram showing solutions as a function of the depletion rate *k*_-_. Stable fixed point (thin solid curve) undergoes a Hop bifurcation at the points H, resulting in an unstable fixed point (dotted curve) and a stable limit cycle (thick solid curve). (b) Trajectories along the limit cycle for *x*(*t*) (solid curve) and *q*(*t*) (grey curve). Parameter values are *k*_+ _= 0.02, *γ *= 20, *F*_0 _= 1 and *h *= -0.15 with *k*_- _= 0.1 in (b).

Suppose that the network operates in a parameter regime where there exists a unique fixed point. By linearizing about the fixed point and calculating the eigenvalues of the Jacobian, we can find regimes where the fixed point destabilizes via a Hopf bifurcation leading to the formation of a limit cycle. An example bifurcation diagram is shown in Figure 9 with the depletion rate *k*_- _treated as the bifurcation parameter. Also shown is an example of a limit cycle for a particular value of *k*_-_. Given a limit cycle solution (x*(*θ*), q*(*θ*)) and associated isochronal function *Ψ *(*x*, *q*), we can numerically calculate the components of the corresponding phase resetting curve, which are defined according to(43)

together with the normalization condition

**Figure 10:**
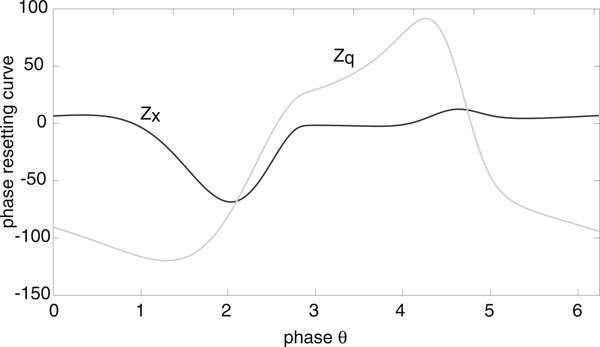
**Components *Z_x _*and *Z_q _*of phase resetting curve for an excitatory network with synaptic depression**. Same parameter values as Fig. 9(b).

The components of the phase resetting curve for the limit cycle shown in Figure 9(b) are plotted in Figure 10. As in the case of the E-I network, the resulting population oscillator is type II. However, the PRC is no longer approximately sinusoidal.

### Stochastic network and noise-induced synchronization

We will now construct a stochastic version of the above population model by assuming that the population activity evolves according to a birth-death master equation with transition rates that depend on the depression variable *q*. (Both the deterministic and stochastic models make a strong simplication by assuming that synaptic depression, which occurs at individual synapses, can be represented in terms of a single scalar variable *q*).^b ^Let *N*(*t*) denote the number of excitatory neurons active at time *t*, with *P*(*n*, *t*) = Prob[*N*(*t*) = *n*] evolving according to the master equation (1) with *M *= 1:(44)

with *P*(-1, *t*) ≡ 0. The transition rates are taken to be of the Wilson-Cowan form(45)

where *I *is an external input, and *q*(*t*) satisfies(46)

The master equation (44) is non-autonomous due to the dependence of the birth rate *T*_+ _on *q*(*t*), with the latter itself coupled to the associated jump Markov process via the depletion rate *k*_-_*X*(*t*). Thus equation (46) is only defined between jumps, during which *q *evolves deterministically.

The system defined by equations (44)-(46) is an example of a so-called stochastic hybrid model based on a piecewise deterministic process. This type of model has recently been applied to genetic networks [55] and to excitable neuronal membranes [56,44,52]. In the latter case, the hybrid model provides a mathematical formulation of excitable membranes that incorporates the exact Markovian dynamics of single stochastic ion channels. Moreover, the limit theorems of Kurtz [48] can be adapted to prove convergence of solutions of the hybrid model to solutions of a corresponding Langevin approximation in the limit *N *→ ∞ and finite time, where *N *is the number of ion channels within the membrane [44,52].

In the case of our stochastic hybrid model of an excitatory network with synaptic depression, we can heuristically derive a Langevin approximation by first carrying out a Kramers-Moyal expansion of the master equation (44). That is, setting *x *= *n*/*N *and treating *x *as a continuous variable, we Taylor expand the master equation to second order in 1/*N *to obtain the Fokker-Planck equation(47)

with *ε *= *N*^-1/2^,(48)

and(49)

The solution to the Fokker-Planck equation (47) determines the probability density function for a corresponding stochastic process *X*(*t*), which evolves according to the Langevin equation(50)

with *b*(*x*, *q*)^2 ^= *B*(*x*, *q*), *W*_1_(*t*) an independent Wiener process and, from equation (46),(51)

Following along similar lines to the *E *- *I *network, we can also include an extrinsic noise source by decomposing the drive to the excitatory population as(52)

where *h *is a constant input and  is a white noise term of strength *σ*. Substituting for *I *in equation (50) and assuming that *σ *is sufficiently small, we can Taylor expand to first order in *σ *to give(53)

where *W *is a second independent Wiener process and(54)

Suppose that we have an ensemble of excitatory networks with synaptic depression labeled *μ *= 1, ..., , each of which supports a stable limit cycle (x*(*θ*), q*(*θ*) in the deterministic limit. Carrying out a stochastic phase reduction along similar lines to that of the E-I network, we obtain the following system of Stratonovich Langevin equations:(55)

for *μ *= 1, ..., . Here(56)

with(57)

**Figure 11:**
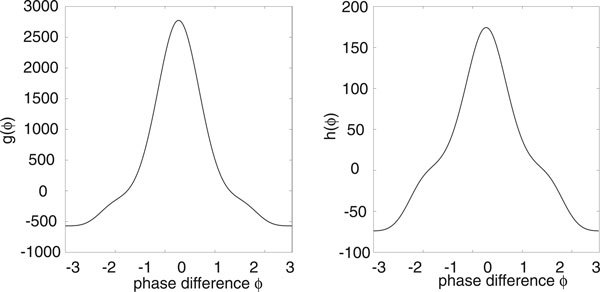
**Plot of periodic functions *g *and *h *for an excitatory network with synaptic depression**. Same parameters as Fig.9(b).

**Figure 12:**
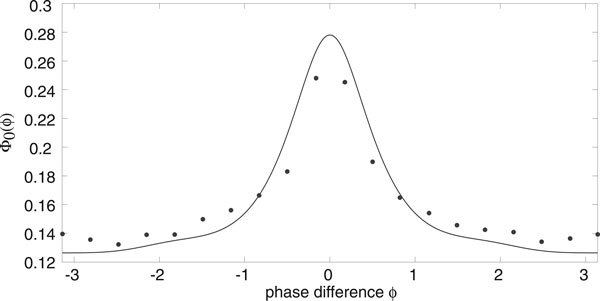
**Probability distribution of the phase difference between a pair of excitatory networks with synaptic depression with *g*, *h *given by Fig. 11**. Solid curves are based on analytical calculations, whereas black filled circles correspond to stochastic simulations of the phase-reduced Langevin equation. Same parameters as Fig. 9(b).

Thus, equations (55) and (56) correspond to equations (27) and (28) for *M *= 1 and *Z*_1_(*θ*) = *Z_x_*(*θ*). However, the functions *Ω*(*θ*), *α*(*θ*), *β*(*θ*) implicitly depend on the dynamics of the depression variable as seen in equation (57). We can now write down the associated Fokker-Planck equation (34) and carry out the averaging procedure of Nakao *et al *[12]. The final result of this analysis is the steady state distribution *Φ*_0_(*ϕ*) for the phase difference *ϕ *of any pair of oscillators given by equation (39) with(58)(59)

and *α*, *β *given by equation (56). An example plot of the periodic functions *g*(*ψ*), *h*(*ψ*) for an excitatory network with synaptic depression is given in Figure 11. In Figure 12 we plot an example of the distribution *Φ*_0 _illustrating how, as in the case of an E-I network, the synchronizing effects of a common extrinsic noise source are counteracted by the uncorrelated intrinsic noise arising from finite-size effects.

## Discussion

In this paper we extended the theory of noise-induced synchronization to a stochastic Wilson-Cowan model of neural population dynamics formulated as a neural master equation. We considered two canonical network structures that are known to exhibit limit cycle oscillations in the deterministic limit; an E-I network of mutually interacting excitatory and inhibitory populations, and an excitatory network with synaptic depression. In both cases, we used phase reduction methods and averaging theory to explore the effects of intrinsic noise on the synchronization of uncoupled limit cycle oscillators driven by a common extrinsic noise source. We achieved this by first approximating the neural master equation by a corresponding neural Langevin equation. Such an approximation is reasonable for sufficiently large system size *N*, and provided that there do not exist other stable attractors of the deterministic system [24]. One important consequence of intrinsic noise is that it broadens the distribution of phase differences. The degree of broadening depends on the term *N*^-1^*h*(0), see equation (39), where *N *is the system size and *h*(0) depends on the intrinsic dynamics of each un-coupled limit cycle oscillator. Another result our study is that the reduction of the master equation generates multiplicative rather than additive terms in the associated Langevin equation for both intrinsic and extrinsic noise sources. Multiplicative noise can lead to clustering of limit cycle oscillators, as was demonstrated in the case of an ensemble of uncoupled E-I networks.

It is important to point out that the master equation formulation of stochastic neurodynamics developed here and elsewhere [21-24] is a phenomenological representation of stochasticity at the population level. It is not derived from a detailed microscopic model of synaptically coupled spiking neurons, and it is not yet clear under what circumstances such a microscopic model would yield population activity consistent with the master equation approach. Nevertheless, if one views the Wilson-Cowan rate equations [18,19] as an appropriate description of large-scale neural activity in the deterministic limit, it is reasonable to explore ways of adding noise to such equations from a top-down perspective. One possibility is to consider a Langevin version of the Wilson-Cowan equations involving some form of extrinsic additive white noise [57,58], whereas another is to view the Wilson-Cowan rate equations as the thermo-dynamic limit of an underlying master equation that describes the effects of intrinsic noise [21-23,20]. As we have highlighted in this paper, the latter leads to a multiplicative rather than additive form of noise.

There are a number of possible extensions of this work. First, one could consider more complicated network architectures that generate limit cycle oscillations at the population level. One particularly interesting example is a competitive network consisting of two excitatory populations with synaptic depression (or some other form of slow adaptation) that mutually inhibit each other. Such a network has recently been used to model noise-induced switching during binocular rivalry [59-64]. Binocular rivalry concerns the phenomenon whereby perception switches back and forth between different images presented to either eye [65,66]. Experimentally, it has been found that the eye dominance time statistics may be t to a gamma distribution, suggesting that binocular rivalry is driven by a stochastic process [67]. One possibility is that there is an extrinsic source of noise associated with the input stimuli. A number of recent models have examined dominance switching times due to additive noise in a competitive Wilson-Cowan model with additional slow adapting variables [61-63]. On the other hand, Laing and Chow [59] considered a deterministic spiking neuron model of binocular rivalry in which the statistics of the resulting dominance times appeared noisy due to the aperiodicity of the high-dimensional system's trajectories. The latter is suggestive of an effective intrinsic noise source within a rate-based population model. A second extension of our work would be to introduce synaptic coupling between the limit cycle oscillators. For example, in the case of E-I networks such coupling could represent intracortical connections between columns in visual cortex [37,38]. The effects of mutual coupling on noise-induced synchronization has been explored within the context of a pair of coupled conductance-based neurons [15]. Finally, the neural master equation has certain similarities to individual-based models in theoretical ecology, in particular, stochastic urn models of predator-prey systems [68,69]. Given that predator-prey systems often exhibit limit cycle oscillations and receive extrinsic environmental signals, it would be interesting to extend our results on neuronal population oscillators to explore the effects of demographic noise on the stimulus-induced synchronization of an ensemble of ecological communities.

## Competing interests

The authors declare that they have no competing interests.

## Endnotes

^a^One could take the number of neurons in each sub-population to be different provided that they all scaled with *N*. For example, one could identify the system size parameter *N *with the mean number of synaptic connections into a neuron in a sparsely coupled network.

^b^In order to relate the population depression variable *q *to what is happening at individual synapses, we label individual neurons within an excitatory network by the index *a *= 1, ..., *N *and assume that the neurons are globally coupled. Suppose that the ring rate *r_a _*of the *a*th neuron evolves according to

Summing the second equation with respect to *b *and dividing through by *N *leads to the following equation for ,

provided that the following mean-field approximation holds.

If we then average the first equation with respect to *a *and again impose the mean field approximation, we see that

Finally, noting that *q_a_*(*t*) → *q*(*t*) for sufficiently large *t *(after transients have disappeared), we recover equations (42). In constructing a stochastic version of the network, we will assume that the above mean-field approximation still holds even though the activity variables are now random. See [70] for a recent discussion of the validity of mean-field approximations in a stochastic network model with synaptic depression.
